# Resolution, quantification, and reliable determination of enantiomeric excess of proteinogenic and non‐proteinogenic amino acids by comprehensive two‐dimensional gas chromatography

**DOI:** 10.1002/jssc.202200606

**Published:** 2022-10-17

**Authors:** Raphaël Pepino, Vanessa Leyva, Adrien D. Garcia, Jana Bocková, Cornelia Meinert

**Affiliations:** ^1^ Institut de Chimie de Nice, CNRS UMR 7272 Université Côte d'Azur Nice France

**Keywords:** amino acid, chirality, enantioseparation, *N*‐trifluoroacetyl‐*O*‐methyl ester, two‐dimensional gas chromatography

## Abstract

This work proposes a comprehensive two‐dimensional gas chromatography method for the resolution and quantification of 27 amino acids, including 17 enantiomeric pairs, as stable *N*‐trifluoroacetyl‐*O*‐methyl ester derivatives. The derivatization approach in combination with enantioselective two‐dimensional gas chromatography has proven to be highly responsive with a method detection limit of 1–7 pg even for sterically hindered amino acids such as α,α‐dialkylated, and *N*‐alkylated amino acids. Accurate determination of the enantiomeric excess was achieved with errors in the range of ±0.5%–2.5% (1σ) at concentrations ≥10^‒6^ M. A thorough study of the mass spectra of the amino acid derivatives allowed the fragmentation pathways to be distinguished, enabling chromatographic peaks to be unambiguously assigned. The proposed method is particularly suited for applications that require the precise determination of enantiomeric excesses such as those concerning the role of d‐amino acid enantiomers in humans, animals, and the environment, as well as for analyses of extraterrestrial samples aimed at understanding the selection of amino acids in stereochemical l‐configuration.

**Article Related Abbreviations**
*ee*
: enantiomeric excess
MDL: method detection limit
MeOH: methanol
TFA/alkyl: *N‐*trifluoroacetyl‐*O*‐alkyl
TFAA: trifluoroacetic acid anhydride

## 1 INTRODUCTION

Amino acids are fundamental peptide constituents of all living systems on Earth, where they are mostly found in their left‐handed configuration. Their d‐counterparts, long considered biologically non‐functional, are now known to play an important role in a number of physiological processes [1, 2, 3, 4, 5, 6, 7]. The difference in natural abundance and biological activity between the d‐ and l‐amino acid enantiomers has stimulated a growing interest in their analyses in various fields of science and technology, including medicine, biotechnology, food, and natural product research, environmental science, and astrochemistry. For example, proteinogenic d‐amino acids are increasingly being evaluated as potential biomarkers of aging and diseases [7, 8, 9, 10] as well as indicators of food authenticity and quality [7, 11]. Optically pure d‐enantiomers of proteinogenic and non‐proteinogenic amino acids have become a powerful tool for improving the pharmacokinetic properties of peptide–drugs [12, 13] along with key synthetic precursors for biomaterials and biosensors [14, 15]. In astrochemistry, l‐excesses of certain amino acids in interstellar bodies can be interpreted as the result of molecular symmetry‐breaking events with important consequences for the emergence of homochirality in the early evolution of life [16, 17, 18, 19]. All these applications require thorough and comprehensive analytical procedures that ensure: (1) low LOQs, (2) sufficient enantiomeric resolution, (3) absence of racemization during the analysis, and (4) careful control of contamination sources.

LC‐based (HPLC and UPLC) and ^1^D GC‐MS techniques are the most widely used methods for the separation and analysis of complex mixtures of chiral amino acids, the former being generally more sensitive and capable of reaching detection limits two to three orders of magnitude lower than the latter [20, 21, 22]. Comprehensive GC×GC, on the other hand, has been much less explored for the chiral analysis of amino acids, despite its robustness and very high selectivity [23, 24, 25]. Indeed, GC×GC has revolutionized analytical separations in several fields, significantly improving the resolution power and detectability of conventional GC due to the addition of a second orthogonal separation dimension and the one‐dimensional analyte band compression during the modulation process that leads to increased signal‐to‐noise ratio [26, 27, 28]. With its high peak capacity and improved sensitivity in full scan mode due to the efficiency of the mass analyzer and an acquisition rate of up to 500 spectra/s, GC×GC enables unambiguous identification and quantification of complex mixtures – often challenging to resolve with 1‐D chromatography.

Enantioselective GC analysis of amino acids requires their conversion to volatile derivatives. Among the various derivatization protocols for the enantioseparation of amino acids, their two‐step conversion to *N‐*trifluoroacetyl‐*O*‐alkyl (TFA/alkyl) esters has proven to be very successful in the analysis of extraterrestrial samples [21], where high precision enantiomeric excess (*ee*) determination at low concentrations is desired. Furthermore, this method has been particularly advantageous for the detection and separation of a wide range of non‐proteinogenic amino acids, including α,α‐dialkylated amino acids [21, 29], which are known to show very low response with other derivatization methodologies due to low reaction yields [24, 25].

To our knowledge, there is only one report on the enantioseparation of amino acids as TFA/alkyl derivatives by GC×GC. This exploratory analysis was, however, limited to the separation of 12 α‐amino acid enantiomers on a Chirasil‐l‐Val column, lacking information on method validation parameters [23]. The aim of the present study was to develop an improved and validated GC×GC–TOF‐MS methodology for the resolution and quantification of 30 proteinogenic and non‐proteinogenic amino acids, including α‐, β‐, γ‐, δ‐, diamino‐, α,α‐dialkylated and *N*‐alkylated amino acids, as *N*‐trifluoroacetyl‐*O*‐methyl ester derivatives on a chiral octakis(3‐*O*‐butanoyl‐2,6‐di‐*O*‐n‐pentyl)‐γ‐cyclodextrin (Lipodex E) column. This stationary phase was selected because of its demonstrated excellent performance and improved enantioseparation as compared to Chirasil‐Val stationary phases for TFA/methyl amino acid derivatives [29, 30, 31]. We focused on determining and discussing the relevant analytical parameters and their associated uncertainties, such as resolution, enantioseparation, detection limits, repeatability, and stability of the derivatives, often absent in the literature despite their importance for assessing the quality and applicability of a quantitative chiral analytical procedure. Finally, we report here for the first time the mass fragmentation patterns and their interpretation of a series of *N*‐trifluoroacetyl‐*O*‐methyl ester amino acid derivatives.

## 2 MATERIALS AND METHODS

### 2.1 Chemicals and Reagents

Thirty amino acids were studied: *proteinogenic amino acids* (glycine, dl‐alanine, dl‐cysteine, dl‐serine, dl‐aspartic acid, dl‐asparagine, dl‐threonine, dl‐glutamic acid, dl‐proline, dl‐valine, dl‐methionine, dl‐leucine, dl‐isoleucine, and dl‐phenylalanine), *α‐non‐proteinogenic amino acids* (dl‐2‐aminobutyric acid, dl‐norvaline, dl‐norleucine, and dl‐*allo*‐isoleucine), *β‐amino acids* (β‐alanine, dl‐3‐aminobutyric acid, dl‐3‐aminoisobutyric acid and dl‐β‐leucine), a *γ‐amino acid* (4‐aminobutyric acid), a *δ‐amino acid* (5‐aminopentanoic acid), *α,α‐dialkylated amino acids* (2‐aminoisobutyric acid and dl‐isovaline), *N*‐alkylated amino acids (sarcosine, and *N*‐ethylglycine), and *diamino acids* (dl‐2,3‐diaminopropanoic acid and dl‐2,4‐diaminobutanoic acid). For most of the study, racemic standards were used for the chiral amino acids, except isovaline and *allo*‐isoleucine, for which only enantiopure standards were available. All amino acid standards, solvents, and reagents employed in this study were purchased from Sigma‐Aldrich, Fluka, or Acros Organics and stored according to the recommended instructions. The purity of the dl‐, d‐, and l‐amino acids was above 98% in all cases. The water used at all stages of the study – tool cleaning and sample processing – was obtained using a Milli‐Q Direct 8 apparatus (18.2 MΩ cm at 25°C, <2 ppb total organic carbon). All glassware was washed several times with ethanol and Milli‐Q water, wrapped in aluminum foil, and then heated at 500°C for 5 h to remove possible organic contaminants. Polytetrafluoroethylene‐lined lids and caps were washed in the same way. Pipette tips and GC×GC vial inserts were used without further cleaning.

An aqueous amino acid stock solution (10^‒4^ M) of 30 amino acids was prepared from individual amino acid solutions of 10^‒1^ or 10^‒2^ M, depending on their solubility in water as well as serial dilutions (5×10^‒5^–5×10^‒‍8^ M) of the stock solution. For the experiments aimed at *ee* determination, two separate stock solutions of five selected amino acids with different retention times and functional groups (Ala, 2,3‐Dap, Glu, Iva, and Pro) were prepared. One solution contained the five amino acids in a racemic ratio, while the second standard mixture was spiked with the respective l‐enantiomers to obtain %*ee*_l_  = 5 for each amino acid. Four dilutions (10^‒7^ M, 10^‒6^ M, 5×10^‒6^ M, and 10^‒5^ M) were prepared to evaluate the effect of concentration on accurate %*ee* measurements.

### 2.2 Derivatization procedure

Amino acids were transformed into *N*‐trifluoroacetyl‐*O*‐methyl ester derivatives based on the procedure reported by Fox et al. for conventional GC‐FID [29], with some modifications. A volume of 50 μl of the aqueous amino acid solution was transferred into a reaction vial and dried under a gentle stream of nitrogen. Then, 200 μl of a methanol/acetyl chloride (MeOH/AcCl) (4:1, *v*/*v*) solution was added and the reaction mixture was vigorously stirred for 10 s and heated at 110°C for 1 h. The mixture was then cooled down for 10 min and dried under nitrogen without succeeding in complete evaporation of the solvent in reasonable timeframes as a compromise to avoid potential analyte loss. Subsequently, 200 μl of a dichloromethane/trifluoroacetic acid anhydride (DCM/TFAA) solution (4:1, *v*/*v*) was added, and the reaction medium was stirred for 10 s and then heated at 100°C for 20 min. The solution was then cooled and subjected to a complete drying step to minimize the negative impact of excessive TFA on the GC columns. Care was taken to use a very gentle stream of nitrogen in this step to avoid the loss of highly volatile compounds such as α,α‐dialkylated amino acid derivatives. Finally, the residue was dissolved in 50 μl of methyl laurate (internal standard, IS) in chloroform (10^–5^ M) and transferred into a 1 ml GC vial equipped with a 100 μl insert for GC×GC analysis.

For assessing the stability of the *N*‐trifluoroacetyl‐*O*‐methyl ester derivatives, one amino acid standard mixture at 5×10^–5^ M was derivatized and analyzed after 7 days. The sample was injected in triplicate each time and stored immediately after each injection at 4°C.

### 2.3 GC×GC–TOF‐MS analysis

The enantioselective analyses were carried out using a GC×GC Pegasus IV D instrument coupled to a reflectron time‐of‐flight mass spectrometer (LECO Corp., St. Joseph, Michigan, USA). The TOF‐MS system operated at a storage rate of 150 Hz, with a 50–‍400 amu mass range, and a solvent delay of 15 min. The detector voltage was set to 1650 V, except for the measurements concerning the determination of the method detection limit (MDL), for which a voltage of 1800 V was used. The ion source and injector temperatures were kept at 230°C and the transfer line at 240°C. The column set consisted of a Lipodex E [octakis(3‐*O*‐butanoyl‐2,6‐di‐*O*‐n‐pentyl)‐γ‐cyclodextrin] capillary column (25 m × 0.25 mm, Macherey‐Nagel, Düren Germany) connected in series to a DB Wax (polyethylene glycol, 1.4 m × 0.1 mm, 0.1 μm, Agilent, CA, USA) secondary column. Alternatively, a CP‐Chirasil‐Dex CB [heptakis‐(2,3,6‐tri‐*O*‐methyl)‐β‐cyclodextrin] column (24.55 m × 0.25 mm, 0.25 μm, Agilent) was used in the first dimension for qualitative comparison of the overall resolution efficiency and enantioseparation of the amino acid standard mixture compared to the Lipodex E. 2‐propanol and chloroform were used as washing solvents for the injection needle. Modulation between columns was ensured with a dual‐stage thermal jet modulator using liquid nitrogen. Helium was used as carrier gas at a constant flow of 1 ml/min. Aliquots of 1 μl were injected in splitless mode.

For the experiments involving the entire set of amino acids including the determination of detection limits, the temperature of the primary column was held at 40°C for 1 min, increased to 80°C at a rate of 10°C/min, and held for 10 min, followed by an increase to 125°C at 1°C/min, and finally to 190°C at 2°C/min and held for 1 min. For the determination of *ee* reliability, which included only 5 of the 30 amino acids under study, the temperature of the primary column was held at 40°C for 1 min, increased to 80°C at a rate of 10°C/min, held for 10 min, and finally increased to 190°C at 2°C/min and held for 5 min. For both types of analyses, the secondary oven was operated at a constant temperature offset of 30°C, while the temperature of the dry air used for the modulator's hot pulses was set at 15°C above the secondary oven temperature and a modulation period of 5 s was applied. Data were processed using the ChromaTOF software from LECO Corp. Integrated peak areas were adjusted manually, when necessary, to correct for automatic data treatment limitations.

### 2.4 LOD and MDL

The LOD is defined as the lowest concentration of an analyte that can be identified and measured with high confidence that the concentration of the analyte is greater than zero. It is a fundamental parameter for assessing the quality and range of applicability of an analytical methodology, especially when low concentrations are used to make decisions [32, 33]. However, the LOD is also an ambiguous and controversial concept, as it can vary up to three orders of magnitude, depending on the approach chosen for its determination and the objective of the measurement, which makes comparisons difficult [33, 34, 35]. In chromatography, LOD is usually estimated as a multiple of the average background signal‐‍to‐‍noise ratio of the signal arising from a reagent blank. However, not only do these approaches rely heavily on the region of the chromatogram selected for noise measurements, but they obviate measuring the analyte itself [36]. This problem is even more evident for methodologies employing MS detectors, for which background noise sources have been significantly reduced and specificity increased, resulting in the near‐zero instrumental background when the analyte of interest is absent [37]. Therefore, for MS‐based chromatographic techniques, multi‐injection approaches based on the SD of the analyte response at a concentration close to the expected detection limit are considered to give more reliable results, as they take into account the actual signal intensities and the consistency of the response over several repeated injections [34, 37]. Accordingly, for the present study, the LODs of amino acids were determined using the MDL approach, which defines the MDL as the minimum amount of analyte that can be measured with 99% confidence that the measured concentration is distinguishable from, greater than, zero, according to the following equation:

(1) $$MDL=t_{\alpha }\times \frac{\mathrm{RSD}}{100}\times Q_{s}$$

where *t*_α_ is the one‐sided Student's t‐distribution value for n‐1 observations at a 99% confidence level (α), RSD corresponds to the relative SD of the peak areas of the *n* replicate injections for each amino acid, and *Q*_S_ is the concentration of the analytes at the expected LOD. As eight replicates (*n* = 8) of the standard solution at *Q*_S_ = 5×10^‒8^ M were employed, *t*_α_ was set to 2.998. The RSD of the measured area response was calculated for each individual amino acid.

### 2.5 Enantiomeric excess determination

The enantiomeric excess (*ee*) reflects the excess in the amount of one enantiomer over the amount of the racemic composition, with values that extend from *ee* = 0, for racemic mixtures, to *ee* = 1 (or ‐1), for a pure enantiomer [38]. The l‐enantiomeric excess of a chiral species can be written as follows

(2) $$\%ee_{L}=\frac{c_{L}-c_{D}}{c_{L}+c_{D}}\times 100$$

where c_L_ and c_D_ are the concentrations of l and d‐enantiomers, respectively. Providing that within the studied concentration range for both enantiomers the concentration c_i_, i Є {l; d}, scales with the corresponding ion peak counts A_i_, in single or total ion chromatograms, as

(3) $$C_{i}=k\times A_{i}$$

where *k* is a constant, we can substitute the concentrations c_L_ and c_D_ in Equation (2) with A_L_ and A_D_,

(4) $$\%ee_{L}=\frac{A_{L}-A_{D}}{A_{L}+A_{D}}\times 100$$

The SD $\sigma_{\%ee_{L}}$ is given based on the calculated %*ee*_L_ of each independent injection using Equation (4).

Although commercial amino acid standards are available as racemic mixtures, their %*ee* determined by chromatographic techniques is usually different from zero [39]. This is due to both sample and instrument artifacts. The former is related to the limitations of the manufacturer's accuracy in determining the *ee* as well as to the initial syntheses. The latter is a result of differential instrument responses for each enantiomer or erroneous chromatographic quantification caused by co‐eluting impurities, poor enantio‐resolution, or peak tailing [40]. Consequently, chromatographic techniques do not necessarily provide accurate information on the absolute configuration of the sample. Here, we investigate the performance of the studied GC×GC method for the determination of apparent %*ee*_L_ of 5% by comparing the response of a racemic standard (S_ref_) with a racemic standard spiked with the respective l‐enantiomers (S_spiked_). The %*ee*_L_ is calculated as follows

(5) $$\%ee_{L}=100\times \left(ee_{L\_\mathrm{spiked}}-ee_{L\_\mathrm{ref}}\right)$$

and the corresponding SD based on the error propagation formula as

(6) $$\sigma_{ee_{L}}=\sqrt{{\sigma_{ee_{L\_\mathrm{spiked}}}}^{2}+{\sigma_{ee_{L\_\mathrm{ref}}}}^{2}}$$

where $\sigma_{ee_{l\_\mathrm{spiked}}}$ and $\sigma_{ee_{l\_\mathrm{ref}}}$ are the SDs of the spiked and racemic standards, respectively. To account for procedural and measurement fluctuations, three individually derivatized samples were prepared and injected in triplicates, for a total of nine injections (*n* = 9) per amino acid in the racemic and spiked solutions.

## 3 RESULTS AND DISCUSSION

### 3.1 Chromatographic resolution

**FIGURE 1:**
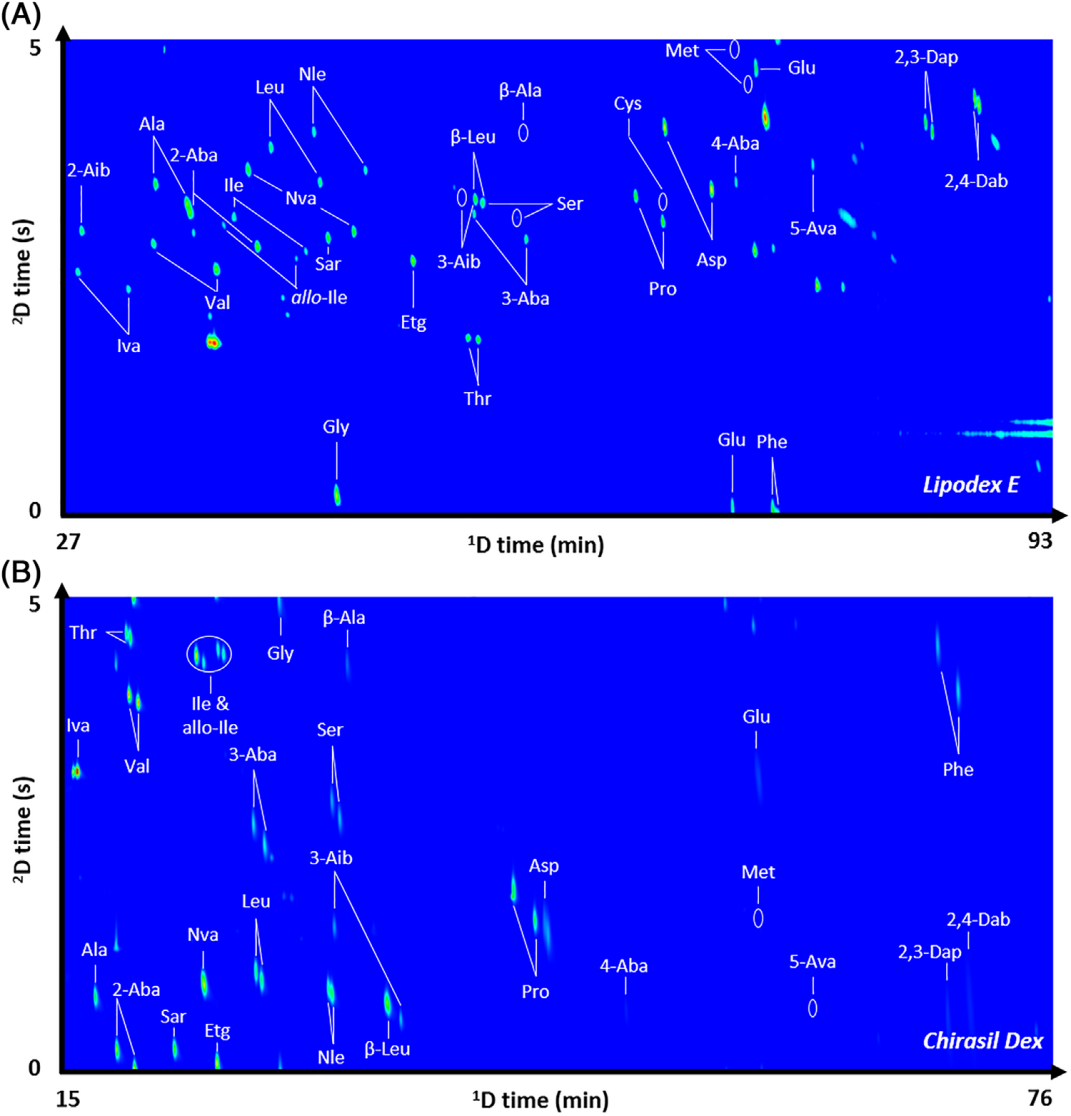
Two‐dimensional gas chromatogram of the *N*‐‍trifluoroacetyl‐*O*‐methyl amino acid ester derivatives resolved on a Lipodex E in the first dimension coupled to a DB Wax in the second dimension (A) as well as on a Chirasil‐Dex CB coupled to DB‐Wax column configuration (B).

The initial optimization of the enantio‐GC×GC separation of 29 *N*‐TFA/methyl amino acid derivatives, including 22 pairs of amino acid enantiomers and seven achiral amino acids was carried out using a Lipodex E stationary phase in the first dimension. Amino acid identification was confirmed by injecting each amino acid standard individually. The enantiomer elution order was mainly based on previous work [29, 31] and confirmed for the following amino acids: Ala, Asp, 2‐Aba, 2,3‐Dap, Glu, Ile, Iva, Leu, Nva, Pro, Ser, Thr, and Val using an excess of the l‐enantiomer. Using a modulation time of 5 s, balancing between sufficient 2D peak slices while minimizing unfavorable wrap‐around in the second dimension, a good overall resolution was achieved for most of the amino acids (Figure 1A), with baseline separation for 16 out of the 22 enantiomeric pairs investigated (Table 1). This includes isovaline enantiomers, known to be challenging to separate being alpha‐dialkylated [41]. The chosen 2D column configuration showed suitable orthogonality, i.e. the amino acid derivatives were effectively distributed throughout the 2D separation space. The advantages offered by two‐dimensional separation are clearly visible for amino acid enantiomers that are difficult or not at all resolved in the first dimension, such as l‐Iva from 2‐Aib, d‐Ala from d‐Val, and d‐Nle from l‐Leu and Sar. This feature is extremely beneficial for complex mixtures such as those corresponding to real samples in which several compounds may share very similar functional groups and thus retention behavior in the first dimension.

**TABLE 1:** Retention times (*t_1_*, *t_2_*), enantiomeric resolution (*R_s_*), and method detection limit (MDL) of *N*‐trifluoroacetyl‐*O*‐methyl amino acid esters. Fragments are listed by decreasing intensity. Fragments employed for quantification are highlighted in bold

#					*t*_1_	*t*_2_		MDL
	Compound	Class	[M]^+•^	Characteristic ions, *m*/*z*	min:s	s	^1^D *R*_s_^a^	(nM; pg)
**2 Carbons**								
1	Glycine	α	*185*	**126**, 69, 78, 56, 59, 88, 50, 106	45:20	0.27	‐	43; 3.9^b^
**3 Carbons**								
2	Sarcosine	α, *N*	*199*	**140**, 69, 60, 78, 74, 90, 102, 199	44:45	2.91	‐	33; 2.9
3	d‐Alanine	α	*199*	**140**, 69, 70, 92, 59, 102, 93, 66	33:10	3.46	2.8	27; 2.4
4	l‐Alanine				35:15	3.27		32; 2.9^b^
5	β‐Alanine	β	*199*	**55**, 69, **139**, 70, 126, 167, 98, 59	57:50	3.99	‐	21; 1.9
6	d‐Cysteine	α	*327*	69, **117**, 59, 96, 61, 138, 129, 70	66:55	3.14	1	ND
7	l‐Cysteine				67:15	3.08		
8	d‐Serine	α	*311*	69, **138**, 59, 110, 139, 153, 70, 96	55:20	3.32	4.2	28; 3.0
9	l‐Serine				57:25	3.12		22; 2.3
10	d‐2,3‐Diaminopropionic acid	α	*310*	69, **153**, 185, 126, 78, 125, 96, 138	84:50	4.10	1.25	45; 4.7
11	l‐2,3‐Diaminopropionic acid				85:15	4.01		44; 4.5
**4 Carbons**								
12	*N*‐Ethylglycine	α, *N*	*213*	**126**, 154, 69, 56, 140, 78, 74, 116	50:25	2.69	‐	31; 3.2
13	2‐Aminoisobutyric acid	α,α	*213*	**154**, 59, 69, 114, 84, 166, 138, 73	28:10	2.97	‐	28; 2.9
14	d‐2‐Aminobutyric acid	α	*213*	**154**, 69, 126, 59, 56, 84, 96, 116	35:25	3.17	6.9	40; 4.1
15	l‐2‐Aminobutyric acid				40:00	2.82		37; 3.8
16	d‐3‐Aminobutyric acid	β	*213*	69, **140**, 59, 70, 153, 74, 156, 102	54:30	3.16	7	45; 4.6^c^
17	l‐3‐Aminobutyric acid				58:00	2.90		18; 1.8
18	d‐3‐Aminoisobutyric acid	β	*213*	69, **88**, 57, 153, 56, 126, 59, 84	54:35	3.32	3.4	23; 2.4
19	l‐3‐Aminoisobutyric acid				54:30	3.16		24; 2.5
20	4‐Aminobutyric acid	γ	*213*	**74**, 69, 126, 59, 57, 182, 56, 78	72:05	3.49	‐	13; 1.3
21	d‐Aspartic acid	α	*257*	**156**, 59, 198, 69, 166, 85, 61,71	67:20	4.04	6.3	41; 5.5
22	l‐Aspartic acid				70:30	3.40		41; 5.4
23	d‐2,4‐Diaminobutanoic acid	α	*324*	**152**, 69, 126, 153, 57, 56, 78, 185	88:10	4.33	NS	36; 4.2
24	l‐2,4‐Diaminobutanoic acid				88:25	4.27		
25	d‐Threonine	α	*325*	69, **152**, 57, 153, 59, 185, 141, 96	54:05	1.88	1.3	28; 3.3
26	l‐Threonine				54:50	1.87		25; 3.0
**5 Carbons**								
27	d‐Isovaline	α, α	*227*	55, 69, **168**, 166, 114, 138, 59, 110	31:20	2.39	5.9	62; 7.2
28	l‐Isovaline				27:55	2.56		61; 7.1
29	d‐Valine	α	*227*	55, **153**, 168, 69, 114, 125, 59, 56	33:00	2.85	7.3	29; 3.4
30	l‐Valine				37:15	2.59		42; 4.9
31	d‐Norvaline	α	*227*	55, **168**, 126, 69, 114, 153, 59, 56	39:20	3.62	12.3	31; 3.6
32	l‐Norvaline				46:30	2.97		43; 5.1
33	d‐Proline	α	*225*	**166**, 69, 96, 71, 128, 68, 53, 167	67:10	3.09	3	24; 2.8
34	l‐Proline				65:25	3.34		24; 2.8
35	5‐Aminopentanoic acid	δ	*227*	55, **126**, 74, 69, 59, 139, 78, 82	77:15	3.68	‐	ND
36	d‐Glutamic acid	α	*271*	**152**, 69, 180, 212, 57, 59, 55, 82	71:50	0.16	3.2	29; 4.2
37	l‐Glutamic acid				73:25	4.66		28; 4.1
38	d‐Methionine	α	*259*	**61**, 69, 153, 75, 185, 59, 152, 62	71:40	4.84	6.5	ND
39	l‐Methionine				72:45	4.53		
**6 Carbons**								
40	d‐Isoleucine	α	*241*	69, **153**, 185, 57, 182, 125, 126, 59	38:25	3.12	8.3	25; 3.3
41	l‐Isoleucine				43:15	2.78		32; 4.2
42	d‐*allo*‐Isoleucine^d^	α	*241*	69, **153**, 185, 57, 182, 125, 126, 59	37:40	3.06	8.4	–
43	l‐*allo*‐Isoleucine				42:35	2.70		28; 3.6
44	d‐Leucine	α	*241*	69, **140**, **182**, 153, 70, 185, 55, 59	40:55	3.83	5.6	36; 4.8
45	l‐Leucine				44:10	3.48		25; 3.3
46	d‐β‐Leucine	β	*241*	**156**, 69, **198**, 55, 59, 166, 139, 70	54:40	3.30	1	43; 5.6
47	l‐β‐Leucine				55:05	3.27		22; 2.9
48	d‐Norleucine	α	*241*	69, **182**, 126, 153, 114, 70, 55, 59	43:45	4:00	7	28; 3.7
49	l‐Norleucine				47:15	3.61		30; 3.9
**9 Carbons**								
50	d‐Phenylalanine	*α*	*275*	**91**, 162, 69, 65, 131, 103, 77, 51	74:40	0.09	NS	23; 3.8
51	l‐Phenylalanine				74:55	0.01		

^a^Enantioresolution *R*_s_ in the first chromatographic dimension (^1^D, Lipodex E) determined at 10^‒6^ M except for cysteine where *R*_S_ was determined by individual injection at 10^‒‍4^ M. ^b^Include possible contamination, see *Supplementary Information*. ^c^Unexpected coelution with an unknown compound in one sample. ^d^Enantiomer unavailable for this investigation. NS, not enantioseparated. ND, not detected at 5×10^‒8^ M.

As was previously reported, in most cases, the d‐enantiomer elutes before the corresponding l‐enantiomer, except for cyclic proline [30] and isovaline [29] where this order is reversed. The increase in resolution of *N*‐TFA/alkyl amino acid enantiomers on the Lipodex E stationary phase with the shift of the methyl group from the α to the β or γ position was confirmed for the isomers of valine derivatives [29].

Highly retained enantiomers, such as phenylalanine and the diamino acids 2,3‐Dap and 2,4‐Dab, on the other hand, displayed poor baseline resolution. Despite efforts to optimize the GC×GC temperature program, the separation of l‐Ala from d‐2‐Aba, as well as l‐3‐Aib from d‐3‐Aba, β‐Leu from d‐Ser, were unsuccessful. Alternatively, coelutions of the analytes of interest can be minimized by replacing the Lipodex E with a Chirasil‐Dex column in the first dimension (Figure 1B). Using the identical temperature program, the derivatives have a shorter elution time of about 15 min on the Chirasil‐Dex. Ala and 2‐Aba were resolved, phenylalanine enantioseparated, and the l‐3‐Aib/d‐3Aba/β‐Leu/D‐Ser zone was well spread, so that 3‐Aba, Ser, and 3‐AiB were resolved and enantioseparated. The complementary nature of these two column sets allows us to almost fully enantioseparate the 22 chiral amino acids investigated here, with the only exceptions being cysteine (*R*_S_ = 1, Lipodex E), β‐leucine (*R*_S_ = 1, Lipodex E), and 2,4‐Dab (not resolved).

### 3.2 Method Validation

The developed method was tested for quantitative analysis in terms of linearity, sensitivity, repeatability, and resolution (Table 1). Linearity was investigated for 4 orders of magnitude with concentrations of amino acids ranging from 5×10^‒8^ M to 5×10^‒5^ M. The calibration curves were plotted as the average of three replicate measurements (i.e., three different derivatized mixtures at each concentration injected once) of the characteristic ion peak area over the mass‐to‐charge (*m*/*z*) = 74 peak area of the internal standard A_AA_/A_IS_ versus the molar concentration of the amino acids, on a logarithmic scale. The parameters characterizing the linearity of the method are detailed in Table S1. For all the amino acids under study, the least square correlation coefficient values (*R* ^2^) were close to one, supporting a linear relationship between both variables and the wide applicability range for the method. Based on the slopes of the regression fits listed in Table S1 it is possible to affirm that the amino acids show similar sensitivities (i.e., change in signal per unit change in the amount of analyte) which means that the derivatization yields are comparable for all the different types of amino acids analyzed. This represents an important advantage over other derivatization protocols such as their conversion into *N*(*O*,*S*)‐ethoxycarbonyl heptafluorobutyl ester derivatives, for which the reaction yields diamino‐, α,α‐dialkylated and *N*‐alkylated amino acids were substantially diminished [25]. However, the high detection limits of the two sulfur‐containing amino acids, cysteine, and methionine, prevented the establishment of the working range and MDL investigations at the selected concentrations for these acids. Moreover, asparagine is converted into aspartic acid under these derivatization conditions [42], which we confirmed by analyzing an individual asparagine standard. Similar observations have been previously reported for the *N*(*O*,*S*)‐ethoxycarbonyl heptafluorobutyl ester derivatization [43]. As the amide function is easily hydrolyzed, converting asparagine to aspartic acid, the sum Asx of Asp and Asn is reported for the calibration curve and repeatability studies (Table S1 and S3). Asn was excluded from the follow‐up investigations, including the MDL.

The LODs, determined by the MDL method, are reported in Table 1, and the errors associated with these measurements are in Table S2. A simple comparison with other methodologies is not straightforward, but it is possible to affirm that most amino acids show an adequate response, with detection limits ranging from 1.3 to 7.2 pg. The reported method is therefore suitable for trace detection. The lower limit of the working range (5×10^‒8^ M) of our method is of the same order of magnitude as the LODs recently reported using UPLC‐HRMS, currently considered the most sensitive chromatography technique [22]. At the same time, the high resolving power and enantioselectivity, which are severely compromised when UPLC‐HRMS is employed [22], are maintained here, as discussed in further detail below.

The repeatability of the derivatization and subsequent GC×GC–TOF‐MS analysis was examined on nine replicate samples at 5×10^‒5^ M to evaluate the reliability of the derivatization methodology, particularly for highly volatile amino acids that might be lost during the drying steps. RSDs for the repeatability studies are reported in Table S3. Most of these values are between 3% and 9%, except for 2,3‐Dap, threonine, and methionine with RSDs above 30%. In general, the repeatability results support that, for most amino acid derivatives, the MeOH/TFAA derivatization is a reliable methodology including highly volatile derivatives such as 2‐Aib and Iva.

Stability studies of the *N*‐trifluoroacetyl‐*O*‐methyl ester derivatives were performed to determine whether the derivatives can be stored over a period of 7 days without significant degradation. As shown in Table S4, overall recoveries were typically in the 80%–100% range. Most of the volatile derivatives have recoveries closer to 80%–90%, while the less volatile ones reach recoveries of 90%–100%. The only exception is methionine with a loss of almost 50%. These results suggest that sulfur‐containing MeOH/TFAA derivatives may be prone to decomposition.

### 3.3 Mass fragmentation of *N*‐TFA/methyl amino acid derivatives

The interpretation of the mass spectra was based on the fragmentation mechanisms previously described for the *N*‐TFA/alkyl derivatives of proteinogenic amino acids [44, 45, 46]. Table 1 summarizes the characteristic *m*/*z* fragments for all amino acid derivatives analyzed. Parent ions [M]^+•^ are not among the major characteristic ions due to the high probability of fragmentation with a propensity to form relatively more stable fragment ions. All derivatives show a characteristic intense peak at *m*/*z* = 69 corresponding to the [CF_3_]^+^ moiety of the TFAA reagent. Some molecular class‐specific patterns were also observed. Most of the α‐amino acids show a prominent peak at *m*/*z* [M‐59], which corresponds to the loss of the esterified carboxylic acid group [COOCH_3_] resulting from α‐cleavage. Interestingly, this prominent peak is missing in the mass spectra of α‐amino acids exhibiting OH, SH, or two NH_2_ groups. Moreover, the loss of the carboxylic acid group followed by the loss of the side chain as the corresponding olefin [CF_3_‐CO‐NH = CH2]^+^ at *m*/*z* = 126 was observed for most α‐amino acids. An alternative common fragmentation pattern results from the loss of the alkoxy group from the ester followed by the loss of the side chain, giving rise to the [CF_3_‐CO‐NH = CH‐CO]^+^ at *m*/*z* = 153. The [M‐97]^+^ fragment, corresponding to the loss of [CF_3_CO], is also very common but does not show a class‐specific pattern. The *m*/*z* = 59 fragment attributed to [COOCH_3_]^+^ is prominent in the mass spectra of almost all amino acids, except for proline and phenylalanine (cyclic amino acids)*, N*‐alkylated, and diamino acids. A detailed interpretation of the mass spectra of all 30 investigated amino acids is given in Table S5.

### 3.4 Enantiomeric excess accuracy and precision

**FIGURE 2:**
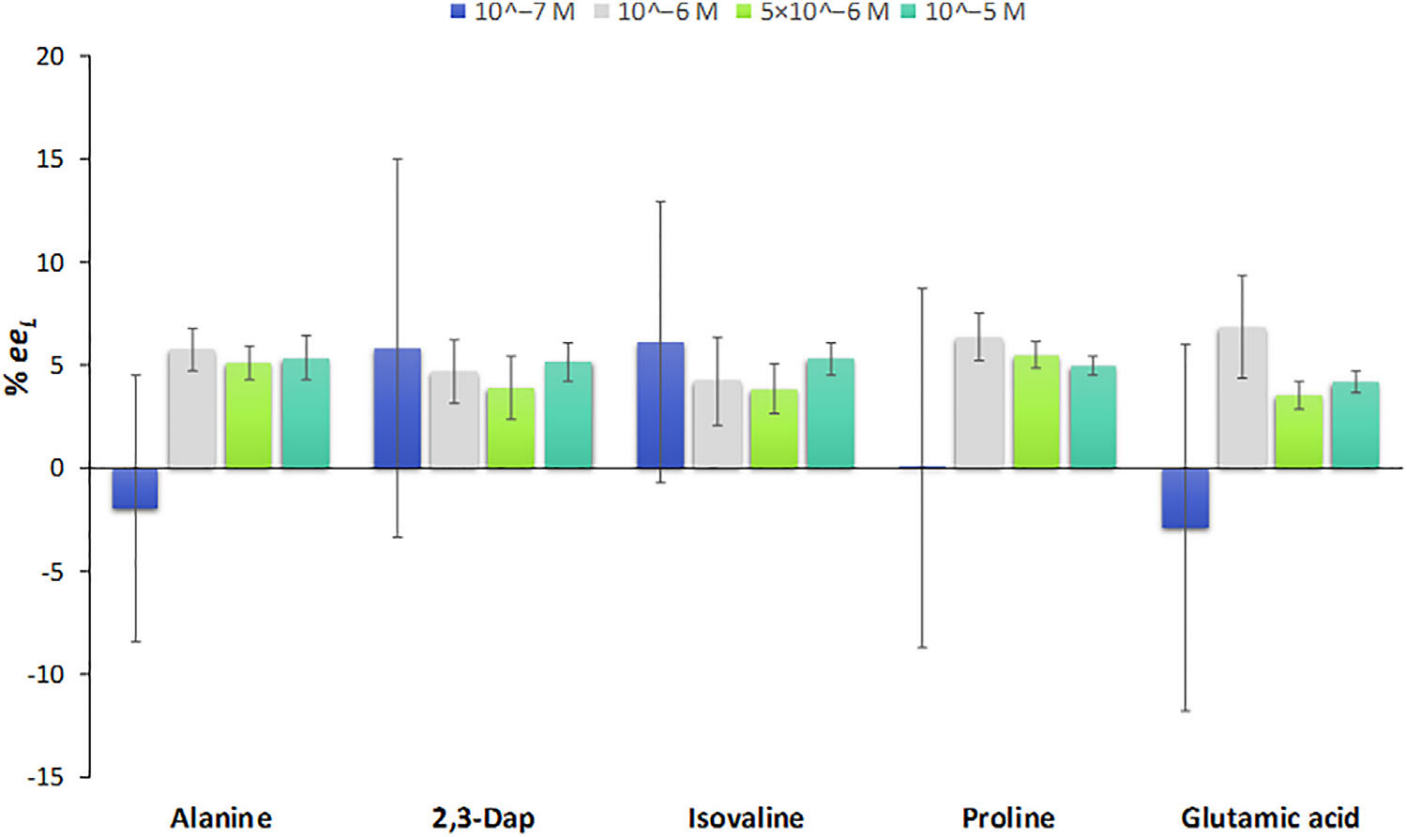
Effect of concentration on the precision of *ee* measurements σ*_ee_*_L_ for five different amino acids spiked with the l‐enantiomer to reach the apparent %*ee*_L_ of 5%. The values represent the average of three replicate samples injected three times (*n* = 9) and uncertainties corresponding to one SD (1*σ*). The bias voltage of the microchannel plate detector was set to 1650 V.

GC×GC–TOF‐MS, often with up to 0.1% (3σ) precision in %*ee* determination, has been considered the gold standard in enantioselective analyses [47]. Notwithstanding, one should keep in mind that the accuracy and precision of %*ee* values determined by GC‐MS are critically affected by the *S*/*N*, enantioseparation, peak broadening, potential co‐elution of one or both enantiomers with other compounds [48] or the detector response. All these parameters can, in principle, vary with the concentration of the enantiomers. To assess the effect of amino acid concentration on the precision of the calculated *ee*, we determined the enantiomeric excess of five amino acids differing in their functional groups (Ala, 2,3‐Dap, Glu, Iva, and Pro) in a solution spiked with the apparent %*ee*_L_ of 5% (Equations 5 and 6, Figure 2), by measuring three replicate samples injected each three times (*n* = 9).

In general, an increase in concentration is associated with a higher *S*/*N* ratio which is, in turn, expected to enhance the precision of the determined *ee*. The effect of the *S*/*N* ratio on the *ee* precision is most pronounced when working close to the LOQ. This is visualized in Figure 2, where changing the concentration by an order of magnitude from 10^‒‍7^ M to 10^‒‍6^ M results in a significant improvement of the SD and consequently in improved accuracy. Increasing the concentration from 10^‒‍6^ M to 10^‒‍5^ M further improves the *ee* precision for 2,3‐Dap, but even more significantly for glutamic acid, isovaline, and proline, which are all well enantioseparated and have narrow peak shapes.

To affirm statistical reliability, and hence high accuracy and precision of calculated enantiomeric excess values, it is important to maximize the number of measurements. This is crucial, especially for measurements with rather low *S*/*N* ratios (10^‒‍7^ M in Figure 2) where the distributions of measured peak areas are broader, and hence undersized sampling may generate skewed results in terms of accuracy but also precision. To enhance the analytical performance at such low concentrations, one can attempt to increase the bias voltage of the microchannel plate detector with the aim to improve the *S*/*N* ratios. In the present study, this has a positive impact on 2,3‐Dap, isovaline, and proline at the concentration 10^‒‍7^ M (Figure S1). Depending on the application, this might be, however, compromised to save the lifetime of the detector.

The precise determination of %*ee* in various applications is often challenged by potential contamination. For example, *proteinogenic* amino acids are susceptible to quantification errors due to contamination from multiple biological sources during sample treatment. A thorough study of reagent blanks was therefore performed to assess such inducible biases (see Supplementary Results S4). Our investigations revealed that l‐proteinogenic amino acids are introduced as minor contaminants (up to 10^–8^ M for Gly, l‐Ala, l‐Val, and l‐Phe) during the derivatization step depending on the analytical grade of TFFA used (Figure S2) and as such constitutes an important exception for trace analyses that need to be monitored. Nevertheless, the careful evaluation of all reagents – especially of TFAA (Table S6) – allowed us to minimize the contribution of l‐amino acids to the order of 10^–11^–10^–10^ M which is orders of magnitudes below the MDL of our method. We, therefore, do not expect any significant bias in the determination of %*ee* in proteinogenic amino acids using the proposed analytical approach.

## 4 CONCLUDING REMARKS

The present study provides a high‐throughput analytical protocol for the enantioseparation of a wide range of chiral amino acids, combining sensitivity, resolution, enantioselective separation, and reliability in the determination of enantiomeric excess over a wide working range (from 5×10^–8^ M to 5×10^–5^ M). This protocol can find numerous applications, from bioanalytical science to extraterrestrial sample analyses. The main advantages of the procedure for enantioselective quantitative analysis are high resolution, increased sensitivity, and low LODs, comparable to or better than those achieved with HPLC and without the corresponding loss in enantioresolution. In addition, the comprehensive mass spectral interpretation of *N*‐trifluoroacetyl‐*O*‐methyl ester derivatives allows unambiguous identification of each amino acid. Finally, our results confirm that concentration is a decisive parameter to be taken into account when assessing the reliability of the reported *ee*s and that, in order to assert statistical reliability and thus high accuracy and precision of the calculated enantiomeric excess values, it is necessary to maximize the number of measurements.

## AUTHOR CONTRIBUTIONS

**Raphaël Pepino**: Investigation, visualization, and writing‐original draft preparation. **Vanessa Leyva**: Investigation and writing‐original draft preparation. **Adrien Garcia**: Investigation. **Jana Bocková**: Investigation, validation, and writing‐ reviewing and editing. **Cornelia Meinert**: Conceptualization, methodology, resources, writing‐original draft preparation, writing‐reviewing and editing, supervision, and funding acquisition.

## CONFLICT OF INTEREST

The authors declare that they have no conflict of interest.

## Supporting information

Supporting Information

## Data Availability

The raw data that support the findings of this study are available from the corresponding author upon reasonable request.
