# Supplementary material for: Resolution, quantification, and reliable determination of enantiomeric excess of proteinogenic and non‐proteinogenic amino acids by comprehensive two‐dimensional gas chromatography
Source: J Sep Sci. 2022 Oct 17;45(24):4416–26. doi: 10.1002/jssc.202200606 (PMC10092029; doi:10.1002/jssc.202200606)
Supplement: Supplementary file 1 — Supporting Information [file JSSC-45-4416-s001.docx]

**Supplementary Information**

**Resolution, quantification, and reliable determination of enantiomeric excess of proteinogenic and non-proteinogenic amino acids by comprehensive two-dimensional gas chromatography**

Raphaël Pepino, Vanessa Leyva, Adrien D. Garcia, Jana Bocková and Cornelia Meinert^∗^

Institut de Chimie de Nice, CNRS UMR 7272, Université Côte d’Azur, 06108 Nice, France

*Corresponding author: cornelia.meinert@univ-cotedazur.fr

This file contains a detailed description of results reported in the main text. It contains Supplementary Figs 1–3 and Supplementary Tables 1–6.

| ***Supplementary Results*** |  |
| --- | --- |
| ***S1: Method Validation*** |  |
| S1.1 Working range & Method detection limit | **2** |
| S1.2 Repeatability and stability of *N*-TFA-*O*-methyl amino acid ester derivatives | **3** |
| ***S2: Mass fragmentation of N-TFA-O-methyl amino acid ester derivatives*** |  |
| S2.1 Mass spectra interpretation | **4** |
| S2.2 Individual mass spectra of 29 *N*-TFA-*O*-methyl amino acid ester derivatives | **5** |
| ***S3 Impact of the detector voltage on enantiomeric excess accuracy and precision*** | **11** |
| ***S4 Assessment of proteinogenic amino acid as contaminants*** | **12** |
| ***Supplementary References*** | **14** |

***S1: Method validation***

***S1.1 Working range & Method detection limit***

**Table S1**: Linear regression data of *N*-trifluoroacetyl-*O*-methyl amino acid esters. The calibration curves were obtained using four concentrations: 5×10^–5^ M, 5×10^–6^ M, 5×10^–7^ M, and 5×10^–8^ M. For each concentration, three samples were prepared and injected once.

| **#** | **Compound** | **S**^a^ | **ΔS**^b^ | **I**^c^ | **ΔI**^d^ | **R^2^** |
| --- | --- | --- | --- | --- | --- | --- |
| 1 | Glycine | 7.79 × 10^4^ | 210 | 3.80 × 10^–2^ | 5.27 × 10^–3^ | 0.99999 |
| 2 | Sarcosine | 7.76 × 10^4^ | 187 | -2.38 × 10^–5^ | 4.70 × 10^–3^ | 0.99999 |
| 3 | d-α-Alanine | 8.69 × 10^4^ | 86.4 | -4.49 × 10^–3^ | 2.17 × 10^–3^ | 0.99999 |
| 4 | l-α-Alanine | 8.74 × 10^4^ | 102 | -2.42 × 10^–3^ | 2.56 × 10^–3^ | 0.99999 |
| 5 | β-Alanine | 2.20 × 10^4^ | 214 | -6.42 × 10^–3^ | 5.37 × 10^–3^ | 0.99981 |
| 6 | d -Cysteine^e^ | ^–^ | ^–^ | ^–^ | ^–^ | ^–^ |
| 7 | l -Cysteine^e^ |  |  |  |  |  |
| 8 | d-Serine | 4.67 × 10^4^ | 634 | -1.98 × 10^–2^ | 1.59 × 10^–2^ | 0.99963 |
| 9 | l-Serine | 4.13 × 10^4^ | 307 | -7.22 × 10^–3^ | 7.71 × 10^-3^ | 0.99989 |
| 10, 11 | d,l-2,3-Diaminopropionic acid^f^ | 5.70 × 10^4^ | 308 | -1.08 × 10^–2^ | 7.75 × 10^-3^ | 0.99994 |
| 12 | *N*-Ethylglycine | 4.69 × 10^4^ | 122 | -4.42 × 10^–3^ | 3.06 × 10^-3^ | 0.99999 |
| 13 | 2-Aminoisobutyric acid | 6.80 × 10^4^ | 60.7 | -3.60 × 10^–3^ | 1.53 × 10^-3^ | 0.99999 |
| 14 | d-2-Aminobutyric acid | 5.80 × 10^4^ | 152 | -5.97 × 10^–3^ | 3.81 × 10^-3^ | 0.99999 |
| 15 | l-2-Aminobutyric acid | 6.28 × 10^4^ | 436 | 8.23 × 10^–3^ | 1.10 × 10^-2^ | 0.99990 |
| 16 | d-3-Aminobutyric acid | 3.50 × 10^4^ | 479 | -1.31 × 10^-2^ | 1.20 × 10^-2^ | 0.99963 |
| 17 | l-3-Aminobutyric acid | 3.25 × 10^4^ | 439 | -1.22 × 10^-2^ | 1.10 × 10^-2^ | 0.99964 |
| 18 | d-3-Aminoisobutyric acid | 2.64 × 10^4^ | 65.8 | -2.40 × 10^-3^ | 1.65 × 10^-3^ | 0.99999 |
| 19 | l-3-Aminoisobutyric acid | 3.33 × 10^4^ | 523 | -1.46 × 10^-2^ | 1.31 × 10^-2^ | 0.99951 |
| 20 | 4-Aminobutyric acid | 2.68 × 10^4^ | 192 | -5.69 × 10^-3^ | 4.82 × 10^-3^ | 0.99990 |
| 21 | d-Aspartic acid + Asparagine^g^ | 5.09 × 10^4^ | 274 | -9.30 × 10^-3^ | 6.87 × 10^-3^ | 0.99994 |
| 22 | l-Aspartic acid + Asparagine^g^ | 4.21 × 10^4^ | 623 | 1.47 × 10^-2^ | 1.56 × 10^-2^ | 0.99956 |
| 23, 24 | d,l-2,4-Diaminobutanoic acid^f,h^ | 9.54 × 10^4^ | 6540 | -1.52 × 10^-1^ | 1.64 × 10^-1^ | 0.99069 |
| 25 | d-Threonine | 3.87 × 10^4^ | 56.3 | -2.67 × 10^-3^ | 1.41 × 10^-3^ | 0.99999 |
| 26 | l-Threonine | 4.58 × 10^4^ | 402 | -1.18 × 10^-2^ | 1.01 × 10^-2^ | 0.99985 |
| 27 | d-Isovaline | 2.57 × 10^4^ | 110 | 1.11 × 10^-3^ | 2.75 × 10^-3^ | 0.99996 |
| 28 | l-Isovaline | 2.99 × 10^4^ | 71.5 | 1.59 × 10^-4^ | 1.80 × 10^-3^ | 0.99999 |
| 29 | d-Valine | 3.30 × 10^4^ | 24.7 | -1.37 × 10^-3^ | 6.21 × 10^-4^ | 0.99999 |
| 30 | l-Valine | 3.26 × 10^4^ | 180 | 3.73 × 10^–3^ | 4.51 × 10^-3^ | 0.99994 |
| 31 | d-Norvaline | 3.72 × 10^4^ | 30.5 | -1.18 × 10^–3^ | 7.66 × 10^-4^ | 0.99999 |
| 32 | l-Norvaline | 3.79 × 10^4^ | 197 | -6.25 × 10^–3^ | 4.94 × 10^-3^ | 0.99995 |
| 33 | d-Proline | 6.60 × 10^4^ | 275 | -9.34 × 10^–3^ | 6.92 × 10^-3^ | 0.99997 |
| 34 | l-Proline | 6.91 × 10^4^ | 326 | -1.05 × 10^–2^ | 8.20 × 10^-3^ | 0.99996 |
| 35 | 5-Aminopentanoic acid | 1.71 × 10^4^ | 177 | -4.95 × 10^–3^ | 4.44 × 10^-3^ | 0.99979 |
| 36 | d-Glutamic acid | 5.78 × 10^4^ | 982 | -2.71 × 10^–2^ | 2.47 × 10^-2^ | 0.99942 |
| 37 | l-Glutamic acid | 5.03 × 10^4^ | 526 | -1.40 × 10^–2^ | 1.32 × 10^-2^ | 0.99978 |
| 38 | d-Methionine^e^ | ^–^ | ^–^ | ^–^ | ^–^ | ^–^ |
| 39 | l-Methionine^e^ |  |  |  |  |  |
| 40 | d-Isoleucine | 2.65 × 10^4^ | 54.8 | 4.06 × 10^–4^ | 1.38 × 10^-3^ | 0.99999 |
| 41 | l-Isoleucine | 2.68 × 10^4^ | 40.6 | 2.32 × 10^–4^ | 1.02 × 10^-3^ | 0.99999 |
| 42 | d-*allo*-Isoleucine | 1.82 × 10^4^ | 105 | -3.54 × 10^–3^ | 2.64 × 10^-3^ | 0.99993 |
| 43 | l-*allo*-Isoleucine | 1.61 × 10^4^ | 52.8 | 6.35 × 10^–4^ | 1.33 × 10^-3^ | 0.99998 |
| 44 | d-Leucine | 2.41 × 10^4^ | 93.1 | -3.29 × 10^–3^ | 2.34 × 10^-3^ | 0.99997 |
| 45 | l-Leucine | 2.40 × 10^4^ | 87.3 | -3.15 × 10^–3^ | 2.19 × 10^-3^ | 0.99997 |
| 46 | d-β-Leucine | 3.43 × 10^4^ | 501 | -1.43 × 10^–2^ | 1.26 × 10^-2^ | 0.99957 |
| 47 | l-β-Leucine | 3.27 × 10^4^ | 647 | -1.82 × 10^–2^ | 1.63 × 10^-2^ | 0.99922 |
| 48 | d-Norleucine | 3.41 × 10^4^ | 340 | -1.03 × 10^–2^ | 8.55 × 10^-3^ | 0.99980 |
| 49 | l-Norleucine | 3.40 × 10^4^ | 449 | -1.26 × 10^–2^ | 1.13 × 10^-2^ | 0.99965 |
| 50, 51 | d, l-Phenylalanine^f^ | 2.11 × 10^5^ | 393 | -1.16 × 10^–2^ | 9.86 × 10^-3^ | 0.99999 |

^a^ Slope. ^b^ Standard error on S. ^c^ Intercept. ^d^ Standard error on I. ^e^ LOQ above working range of this study. ^f^ Combined peak area of d- and l-enantiomers due to poor enantioresolution. ^g^ Sum Asx due to hydrolysis of Asn to Asp. ^h^ Does not include the lowest concentration at 5×10^–8^ M.

**Table S2:** Relative standard deviation of 8 samples analyzed once using 5×10^–8^ M of each amino acid for the determination of the method detection limit (MDL).

| **Amino acid** | **RSD^a^ (%)** | **Amino acid** | **RSD^a^ (%)** | **Amino acid** | **RSD^a^ (%)** | **Amino acid** | **RSD^a^ (%)** |
| --- | --- | --- | --- | --- | --- | --- | --- |
| Gly | 29.0 | d-2-Aba | 26.5 | d-Leu | 24.2 | dl-2,4-Dab | 23.7 |
| Sar | 22.0 | l-2-Aba | 24.7 | l-Leu | 16.8 | d-Thr | 18.4 |
| d-α-Ala | 18.2 | d-3-Aib | 15.4 | d-Nle | 18.6 | l-Thr | 16.7 |
| l-α-Ala | 21.4 | l-3-Aib | 16.3 | l-Nle | 20.1 | d-Iva | 41.3 |
| β-Ala | 13.9 | d-Glu | 19.2 | d-β-Leu | 28.7 | l-Iva | 40.5 |
| d-Ser | 18.8 | l-Glu | 18.4 | l-β-Leu | 14.5 | d-Val | 19.2 |
| l-Ser | 14.8 | d-Met | ND | dl-Phe | 15.4 | l-Val | 27.8 |
| d-2,3-Dap | 30.0 | l-Met | ND | d-3-Aba^c^ | 29.7 | d-Nva | 20.7 |
| l-2,3-Dap | 29.1 | d-Ileu | 16.9 | l-3-Aba | 11.8 | l-Nva | 29.0 |
| Etg | 20.4 | l-Ileu | 21.2 | 4-Aba | 8.4 | d-Pro | 16.1 |
| 2-Aib | 18.9 | d-*allo*-Ileu^b^ | – | d-Asp | 27.5 | l-Pro | 16.2 |
|  |  | l-*allo*-Ileu | 18.4 | l-Asp | 27.3 | 5-Ava | ND |

^a^ Relative standard deviation. ^b^ Enantiomer unavailable for this investigation. ^c^ Unexpected coelution with unknown compound in one sample. ND – Not detected at 5×10^‒8^ M.

***S1.2 Repeatability and stability of N-TFA-O-methyl amino acid ester derivatives***

**Table S3:** Repeatability of peak areas of a 9-times individually derivatized amino acid standard mixture at 5×10^‒5^ M.

| **Amino acid** | **RSD^a^ (%)** | **Amino acid** | **RSD^a^ (%)** | **Amino acid** | **RSD^a^ (%)** | **Amino acid** | **RSD^a^ (%)** |
| --- | --- | --- | --- | --- | --- | --- | --- |
| Gly | 4.28 | d-2-Aba | 4.17 | d-Leu | 4.98 | dl-2,4-Dab | 6.84 |
| Sar | 4.95 | l-2-Aba | 5.15 | l-Leu | 2.93 | d-Thr | 34.85 |
| d-α-Ala | 5.08 | d-3-Aib | 4.99 | d-Nle | 4.40 | l-Thr | 38.45 |
| l-α-Ala | 4.42 | l-3-Aib | 6.13 | l-Nle | 5.16 | d-Iva | 5.99 |
| β-Ala | 5.13 | d-Glu | 6.75 | d-β-Leu | 5.23 | l-Iva | 5.98 |
| d-Ser^b^ | – | l-Glu | 4.43 | l-β-Leu | 5.90 | d-Val | 4.51 |
| l-Ser^b^ | – | d-Met | 118.54 | dl-Phe | 5.25 | l-Val | 4.15 |
| d-2,3-Dap^c^ | 38.14 | l-Met | 114.07 | d-3-Aba | 5.86 | d-Nva | 4.20 |
| l-2,3-Dap^c^ | 31.6 | d-Ileu | 4.45 | l-3-Aba | 4.90 | l-Nva | 5.16 |
| Etg | 4.84 | l-Ileu | 4.34 | 4-Aba | 6.67 | d-Pro | 5.91 |
| 2-Aib | 4.94 | d-*allo*-Ileu | 4.83 | d-Asx^d^ | 3.95 | l-Pro | 4.99 |
|  |  | l-*allo*-Ileu | 4.71 | l-Asx^d^ | 3.69 | 5-Ava | 8.93 |

^a^ Relative standard deviation. ^b^ Enantiomer unavailable for this study. ^c^ Temperature program different from the one described in section 2.3 allowing the enantioseparation of 2,3-Dap: the temperature of the primary column was held at 40 °C for 1 min, increased to 80 °C at a rate of 10 °C min^−1^ and held for 10 min, followed by an increase to 150 °C at 1.5 °C min^−1^, and finally to 185 °C at 4 °C min^−1^ and held for 6 min. ^d^ Sum Asx due to hydrolysis of Asn to Asp.

**Table S4**. Recovery tests of *N*-trifluoroacetyl-*O*-methyl amino acid esters after 7 days at 5×10^–5^ M.

| **Amino acid** | **R^a^ (%) ± (%)^b^** | **Amino acid** | **R^a^ (%) ± (%)^b^** | **Amino acid** | **R^a^ (%) ± (%)^b^** | **Amino acid** | **R^a^ (%) ± (%)^b^** |
| --- | --- | --- | --- | --- | --- | --- | --- |
| Gly | 85.0 ± 2.0 | d-2-Aba | 82.8 ± 3.6 | d-Leu | 86.5 ± 3.4 | dl-2,4-Dab | 103.8 ± 6.3 |
| Sar | 73.1 ± 2.6 | l-2-Aba | 82.7 ± 3.5 | l-Leu | 79.1 ± 2.2 | dl-Thr | 91.2 ± 1.6 |
| d-α-Ala | 80.2 ± 3.6 | d-3-Aib | 93.1 ± 2.6 | d-Nle | 89.3 ± 3.7 | d-Iva | 80.3 ± 4.5 |
| l-α-Ala | 82.2 ± 3.9 | l-3-Aib | 88.3 ± 1.7 | l-Nle | 88.1 ± 3.0 | l-Iva | 79.8 ± 5.2 |
| β-Ala | 97.8 ± 1.7 | d-Glu | 99.4 ± 1.1 | dl-β-Leu | 86.8 ± 8.4 | d-Val | 86.6 ± 4.2 |
| d-Ser | 99.8 ± 14.5 | l-Glu | 99.4 ± 1.7 | dl-Phe | 100.5 ± 1.2 | l-Val | 82.5 ± 4.0 |
| l-Ser | 110.1 ± 17.7 | d-Met | 52.4 ± 4.5 | d-3-Aba | 80.1 ± 5.3 | d-Nva | 86.4 ± 3.9 |
| d-2,3-Dap | 104.1± 5.0 | l-Met | 56.3 ± 5.3 | l-3-Aba | 87.6 ± 1.6 | l-Nva | 86.7 ± 2.6 |
| l-2,3-Dap | 97.7 ± 4.2 | d-Ileu | 88.0 ± 3.1 | 4-Aba | 97.9 ± 1.3 | d-Pro | 90.7 ± 1.9 |
| Etg | 86.5 ± 2.6 | l-Ileu | 93.7 ± 3.0 | d-Asp | 97.2 ± 1.7 | l-Pro | 89.6 ± 2.3 |
| 2-Aib | 77.6 ± 4.6 | l-*allo*-Ileu | 82.2 ± 2.3 | l-Asp | 97.4 ± 1.4 | 5-Ava | 98.9 ± 2.1 |

^a^ Recoveries expressed as the ratio of normalized peak areas between samples at *t*_1_ = day 7 over *t*_0_ = 0 day.  ^b^ Relative standard deviations (1σ) determined from one sample injected three times at *t*_0_, and three times at *t*_1_, considering error propagation.

***S2: Mass fragmentation of N-TFA-O-methyl amino acid ester derivatives***

***S2.1 Mass spectra interpretation***

Common fragments of *N*-TFA/alkyl amino acid derivatives are *m/z* = 69 and *m/z* = 59 corresponding to the α-cleavage of the trifuoroacetylated carbonyl group (highlighted in red in **Table S5**) and the ester’s carbonyl (highlighted in blue in **Table S5**), respectively.

The primary amide functions are generally difficult to cleave contrary to secondary amide functions in *N‑*ethylglycine and proline for example.

The ester bond is frequently fragmented leading to a stabilized -C≡O^+^ ion or can lead to a cycle due to the nitrogen attack on the ester’s carbonyl. Both fragmentation pathways have an *m/z* difference of 1 as the hydrogen bonded to the nitrogen is removed. The ester bond cleavage is observed for 3-Aib, 4-Aba and 5-Ava for example.

McLafferty rearrangement competes with α-cleavage of the carbonyls whenever possible to take place. Both carbonyls lead to McLafferty rearrangement, but the ester’s carbonyl McLafferty rearrangement is more likely to be observed.

Some amino acids show unusual *m/z* = 78 and *m/z* = 106 fragments. Both fragments arise from the further decomposition of the *m/z* = 126 fragment as detailed by Manhas *et al*. [1] and are presented hereafter: *m/z* = 106 is obtained after HF loss from *m/z* = 126 and *m/z* = 78 is obtained after CO loss from *m/z* = 106.

***S2.2 Individual mass spectra of 29 N-TFA-O-methyl amino acid ester derivatives***

**Table S5:** Individual mass spectra of the *N*-trifluoroacetyl methyl ester derivatives of the 29 amino acids studied and the interpretation of their most prominent mass fragments**.**

| **(1) Glycine (Gly)** | Molecular ion: 185 |
| --- | --- |
|  |  |
| **(2) Sarcosine (Sar**) | Molecular ion: 199 |
|  |  |
| **(3,4) Alanine (Ala)** | Molecular ion: 199 |
|  |  |
| **(5) β-Alanine (β-Ala)** | Molecular ion: 199 |
|  |  |
| (**6,7) Cysteine (Cys)** | Molecular ion: 327 |
|  |  |

**Table S5**. *Continued*.

| **(8,9) Serine (Ser)** | Molecular ion: 311 | |
| --- | --- | --- |
|  |  | |
| **(10,11) 2,3-Diaminopropionic acid (2,3-Dap)** | | Molecular ion: 310 |
|  | |  |
| **(12) *N*-Ethylglycine (Etg)** | | Molecular ion: 213 |
|  | |  |
| **(13) Aminoisobutyric acid (2-Aib)** | | Molecular ion: 213 |
|  | |  |
| **(14,15) 2-Aminobutyric acid (2-Aba)** | | Molecular ion: 213 |
|  | |  |

**Table S5**. *Continued*.

| **(16,17) 3-Aminobutyric acid (3-Aba)** | Molecular ion: 213 |
| --- | --- |
|  |  |
| **(18,19) 3-Aminoisobutyric acid (3-Aib)** | Molecular ion: 213 |
|  |  |
| **(20) 4-Aminobutyric acid (4-Aba)** | Molecular ion: 213 |
|  |  |
| **(21,22) Aspartic acid (Asp)** | Molecular ion: 257 |
|  |  |

**Table S5**. *Continued*.

| **(23,24) 2,4-Diaminobutanoic acid (2,4-Dab)** | Molecular ion: 324 | |
| --- | --- | --- |
|  |  | |
| **(25,26) Threonine (Thr)** | Molecular ion: 325 |  |
|  |  |  |
| **(27,28) Isovaline (Iva)** | Molecular ion: 227 |  |
|  |  |  |
| **(29,30) Valine (Val)** | Molecular ion: 227 |  |
|  |  |  |

**Table S5**. *Continued*.

| **(31,32) Norvaline (Nva)** | Molecular ion: 227 | | |
| --- | --- | --- | --- |
|  |  | | |
| **(33,34) Proline (Pro)** | Molecular ion: 225 | | |
|  |  | | |
| **(35) 5-Aminopentanoic acid (5-Ava)** | | Molecular ion: 227 |  |
|  | |  |  |
| **(36,37) Glutamic acid (Glu)** | | Molecular ion: 271 |  |
|  | |  |  |

**Table S5**. *Continued*.

| **(38,39) Methionine (Met)** | | Molecular ion: 259 | |
| --- | --- | --- | --- |
|  | |  | |
| **(40,41) Isoleucine (Ile) & (42,43) *allo*-Isoleucine (*allo*-Ile)** | | Molecular ion: 213 | |
|  | |  | |
| **(44,45) Leucine (Leu)** | | Molecular ion: 241 | |
|  | |  | |
| **(46,47) β-Leucine (β-Leu)** | Molecular ion: 241 | |  |
|  |  | |  |

**Table S5**. *Continued*.

| (**48,49) Norleucine (Nle)** | Molecular ion: 241 |
| --- | --- |
|  |  |
| **(50,51) Phenylalanine (Phe)** | Molecular ion: 275 |
|  |  |

***S3: Impact of the detector voltage on enantiomeric excess accuracy and precision***


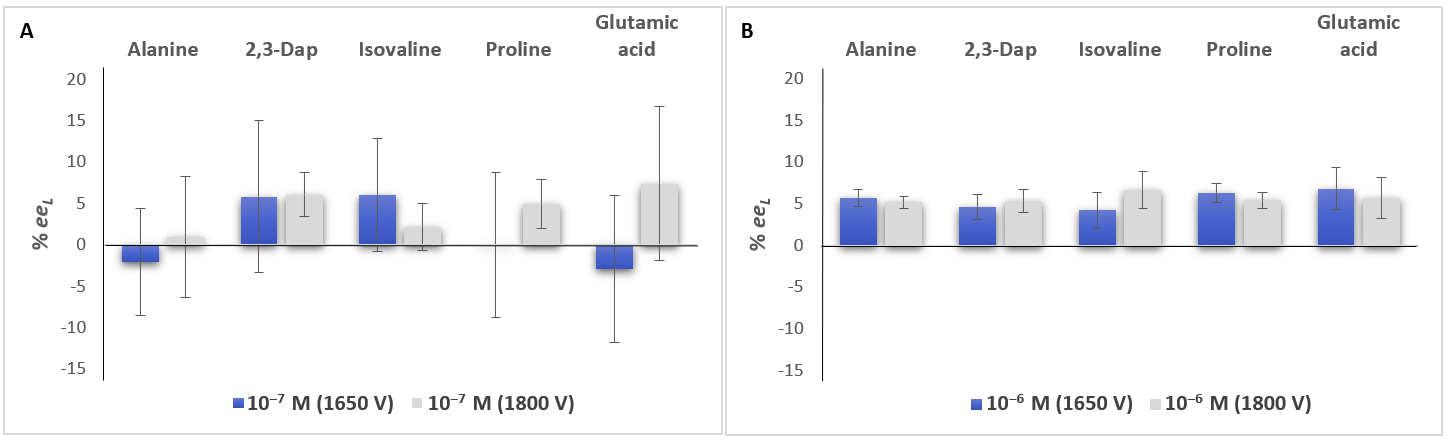


**Figure S1:** Effect of increasing the bias voltage of the microchannel plate detector from 1650 to 1800 V on the %*ee* and the associated standard deviations (± 1σ) at (**A**) 10^‒‍7^ M and (**B**) 10^‒‍6^ M.

***S4 Assessment of proteinogenic amino acid as contaminants***

In this study, the original experiments performed to determine the limit of detection revealed a significant enantiomeric excess of the l-enantiomer of several proteinogenic amino acids including alanine, valine and phenylalanine as well as achiral glycine. This led to the conclusion that considerable contamination was very likely. Free and peptide-bound amino acids were examined in all reagents used including methanol, acetyl chloride, dichloromethane and TFAA by *N*(*O*,*S*)-ethoxycarbonyl heptafluorobutyl ester (ECHFBE) derivatization. The l-amino acid contaminants were detected as complete MeOH/TFAA derivatives, so the first step reagents, methanol (MeOH) and acetyl chloride (AcCl), were studied first. 2 mL of each reagent were dried and derivatized for free amino acids. In addition, 2 mL of each reagent were dried and then hydrolyzed for 1 hour with a 200 µL solution of H_2_O/AcCl (4:1, *v*/*v*) at 110 °C and then derivatized to look for signatures of released proteinogenic amino acids. H_2_O/AcCl, as well as MeOH/AcCl, form hydrochloric acid (HCl) *in situ* which is required for the first esterification step of the MeOH/TFAA derivatization. A procedural blank of the ECHFBE derivatization confirmed the absence of any l-amino acids (**Fig. S2**) or, if present, to be below the instrument’s detection limit.

Surprisingly, MeOH and AcCl did not reveal any contamination with free or peptide-bound amino acids after derivatization (**Fig. S2**). The same study carried out for DCM and TFAA revealed that hydrolyzed TFAA contained a significant excess of l-amino acid, while unhydrolyzed TFAA showed no traces of amino acids. As expected, non-proteinogenic amino acids such as *N*-ethylglycine or isovaline were not detected.

It is interesting to note that it is in the second step of the MeOH/TFAA derivatization (20 minutes at 100 °C) that proteinogenic amino acids such as alanine (**Fig. S2**) are released, for which, however, acidic conditions are required. Since the reaction medium in the first step is difficult to evaporate completely – containing methanol, acetyl chloride, HCl and methyl acetate – it is likely that small traces of the remaining HCl hydrolyze the potential peptide contamination in TFAA. An alternative explanation is the *in situ* formation of trifluoroacetic acetic from TFAA, which acts as an acidic hydrolysis agent.

| 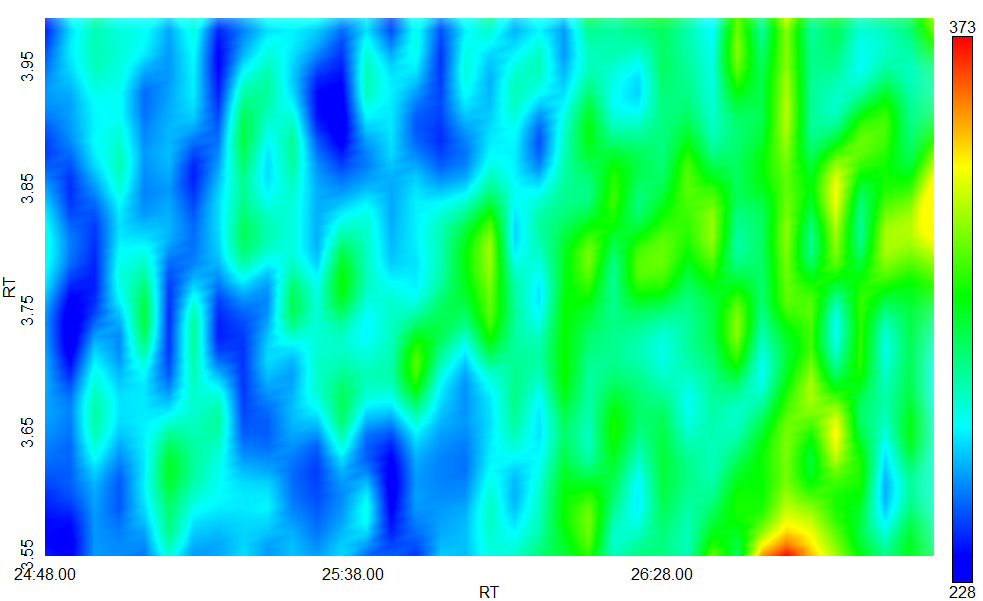  **Blank** (max intensity = 373) | 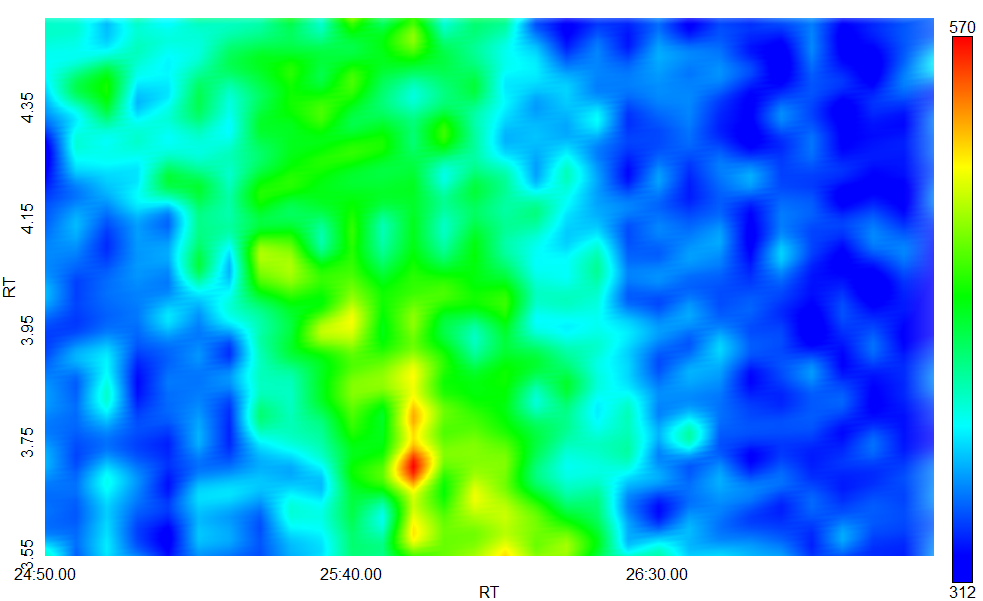  **MeOH** (max intensity = 570) | 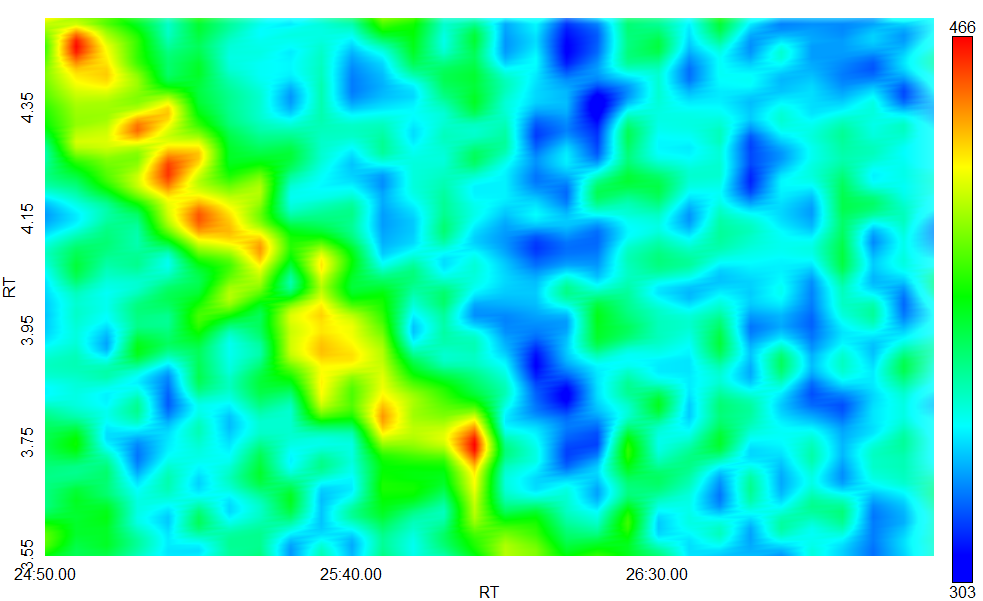  **AcCl** (max intensity = 466) |
| --- | --- | --- |
| 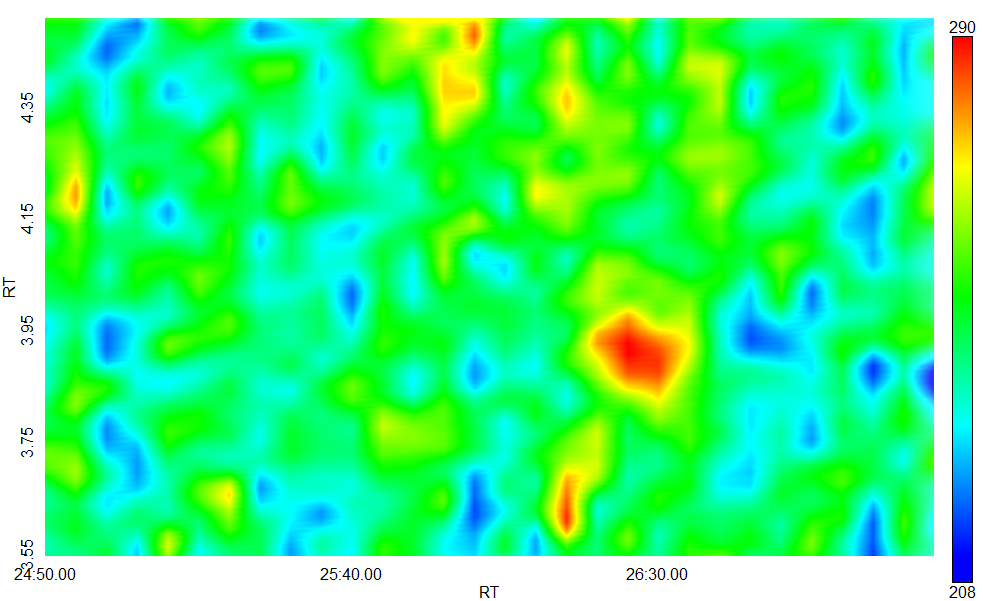  **DCM** (max intensity = 290) | 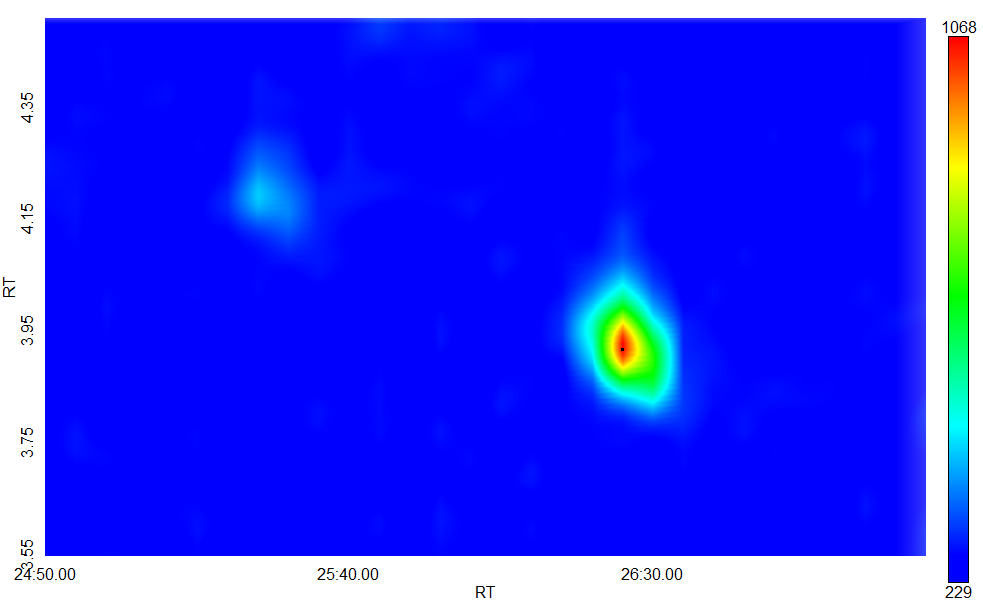  **TFAA** (max intensity = 1068 | 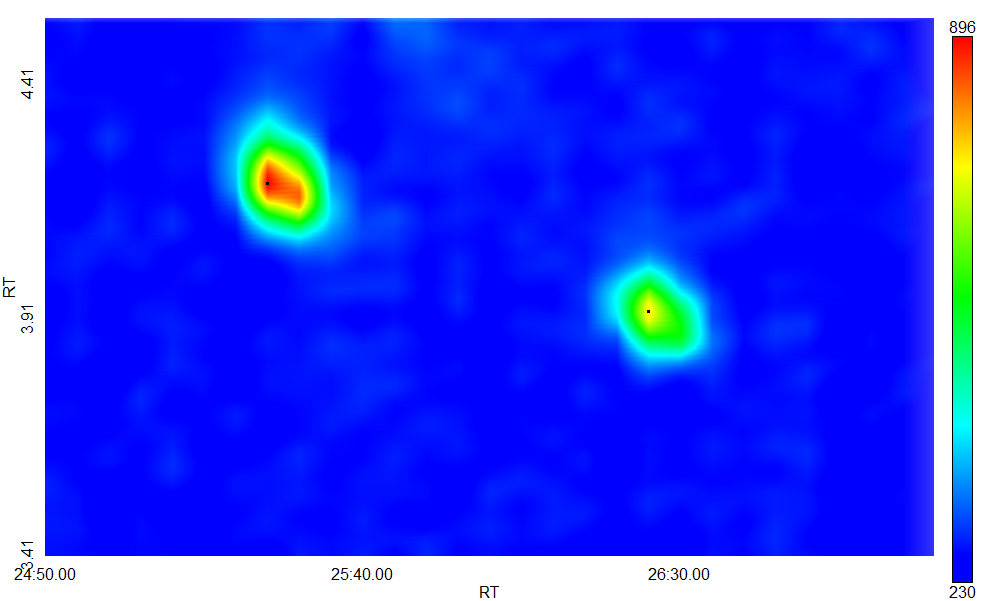  **10^-7^ M dl-Ala** (max intensity = 896) |

**Figure S2:** Evaluation of amino acid contamination using 2 mL dried reagents, hydrolyzed with H_2_0/AcCl and derivatized as ECHFBE derivatives. Fragment ion *m*/*z* = 166 corresponding to the most intense alanine fragment is displayed. A procedural blank and a 10^–7^ M ECHFBE-derivatized dl-alanine sample are provided.

*Quantification of alanine contamination in TFAA*

We estimated the alanine contamination in TFAA by establishing a calibration curve of alanine ECHFBE (**Fig. S3)**. Five standards with concentrations of 10^–5^, 5×10^–6^, 10^–6^, 5×10^–7^ and 2×10^–7^ M of each alanine enantiomer were measured. Three different purity grades of TFAA were investigated and analyzed at a high detector voltage of 1800 V. A few milliliters of each TFAA reagent were dried, hydrolyzed as described above and derivatized with ECHFBE. The calibration curve is shown below and includes the hydrolyzed TFAA_1_ reagent as an example. The important information is the order of magnitude of the contamination, which is between 10^–8^ to 10^–10^ M depending on the TFAA grade studied (**Table S6**). The lowest contamination is obtained when using 1mL-ampules of TFAA.


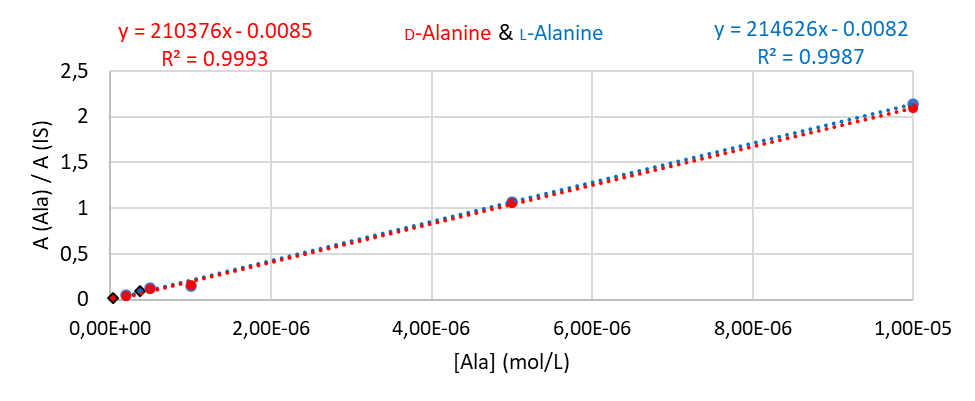


**Figure S3:** Calibration curve of d-Alanine and l-Alanine using ECHFBE derivatization. The small rhombus represents the concentration of alanine found in the TFAA_1_ sample, 3.7×10^–7^ M.

**Table S6**. Evaluation of alanine contamination in three different grades of TFAA.

|  | [d-Ala] (M) | [l -Ala] (M) | Reference sample |
| --- | --- | --- | --- |
| TFAA_1_ | 6.7×10^–10^ | 5.5×10^–9^ | 106232 – 25 g |
| TFAA_2_ | 5.3×10^–9^ | 1.6×10^–8^ | 91719 – 10 × 1 mL |
| TFAA_3_ | ND | 1.4×10^–10^ | 106232 – 10 × 1 g |

As several milliliters were dried and analyzed as a 69 µL sample (50 µL chloroform + 19 µL 2,2,3,3,4,4,4-heptafluorobutanol), the real concentration of l-alanine in TFAA_1_ is calculated through the following equation:

$\text{[L-Ala]}_{\text{TFAA}_{\text{1}}}\text{=} \frac{\text{[L-Ala]}_{\text{TFAA}}^{\text{sample}}\text{ ×} \text{V}_{\text{Analyzed}}}{\text{V}_{\text{TFAA}}}\text{=} \frac{\text{3.7 ×} \text{10}^{\text{-7}}\text{× 0.069}}{\text{4.7}}\text{=}\text{ }\text{5.5}\text{ }\text{×}\text{ }\text{10}^{\text{-}\text{9}}\text{ }$M

This contamination report is only an estimate of the actual contamination because some of the concentrations were calculated by extrapolation to concentrations below the measured calibration curve estimating a linear working range. The presence of other proteinogenic amino acids such as glycine, phenylalanine, glutamic acid, isoleucine, leucine, aspartic acid, and proline was confirmed, especially in the most contaminated TFAA_1_. The concentration of proteinogenic amino acids in TFAA_3_, however, decreased drastically compared to the other TFAA reagents and only minor traces of glycine, phenylalanine, and aspartic acid were found.

***Supplementary References***

[1] Manhas, M. S., Hsieh, R. S., Bose, A. K., Mass spectral studies. Part VII. Unusual fragmentation of some *N*-trifluoroacetyl amino-acid methyl esters. *J. Chem. Soc. C* 1970;116–119.
